# Eukaryotic initiation factor 2 signaling behind neural invasion linked with lymphatic and vascular invasion in pancreatic cancer

**DOI:** 10.1038/s41598-021-00727-3

**Published:** 2021-10-27

**Authors:** Taiichi Wakiya, Keinosuke Ishido, Norihisa Kimura, Hayato Nagase, Tadashi Yoshizawa, Satoko Morohashi, Hiroaki Fujita, Taishu Kanda, Yota Tatara, Junji Saruwatari, Hiroshi Kijima, Kenichi Hakamada

**Affiliations:** 1 Department of Gastroenterological Surgery, Hirosaki University Graduate School of Medicine, 5, Zaifu-cho, Hirosaki, Aomori 036-8562 Japan; 2 Department of Pathology and Bioscience, Hirosaki University Graduate School of Medicine, Hirosaki, Aomori 036-8562 Japan; 3 Department of Stress Response Science, Center for Advanced Medical Research, Hirosaki University Graduate School of Medicine, Hirosaki, Aomori 036-8562 Japan; 4 Division of Pharmacology and Therapeutics, Graduate School of Pharmaceutical Sciences, Kumamoto University, Kumamoto, Kumamoto 862-0973 Japan

**Keywords:** Cancer, Diseases, Gastroenterology, Oncology, Pathogenesis

## Abstract

Perineural invasion (PNI) is a typical poor prognostic factor in pancreatic ductal adenocarcinoma (PDAC). The mechanisms linking PNI to poor prognosis remain unclear. This study aimed to clarify what changes occurred alongside PNI in PDAC. A 128-patient cohort undergoing surgery for early-stage PDAC was evaluated. Subdivided into two groups, according to pathological state, a pancreatic nerve invasion (ne) score of less than three (from none to moderate invasion) was designated as the low-grade ne group. The high-grade (marked invasion) ne group (74 cases, 57.8%) showed a higher incidence of lymphatic metastasis (P = 0.002), a higher incidence of early recurrence (P = 0.004), decreased RFS (P < 0.001), and decreased DSS (P < 0.001). The severity of lymphatic (r = 0.440, P = 0.042) and venous (r = 0.610, P = 0.002) invasions was positively correlated with the ne score. Tumors having abundant stroma often displayed severe ne. Proteomics identified eukaryotic initiation factor 2 (EIF2) signaling as the most significantly enriched pathway in high-grade ne PDAC. Additionally, EIF2 signaling-related ribosome proteins decreased according to severity. Results showed that PNI is linked with lymphatic and vascular invasion in early-stage PDAC. Furthermore, the dysregulation of proteostasis and ribosome biogenesis can yield a difference in PNI severity.

## Introduction

Pancreatic ductal adenocarcinomas (PDAC) has the poorest prognosis of all the world’s cancers^1,2^. Perineural invasion (PNI) is a typical poor prognostic factor in PDAC. Perineural invasion (PNI), including the invasion of extrapancreatic nerve plexus and intrapancreatic nerves, has been characterized by the neoplastic invasion of tumor cells into or surrounding the nerves^3–7^. The prevalence of PNI in PDAC is far higher than in other gastrointestinal malignancies^3,8–11^. Furthermore, the severity of PNI is more pronounced compared to other gastrointestinal malignancies^8^. It has been associated with lymph node metastasis, distant metastasis, tumor recurrence, and poor prognosis in PDAC^12–18^. Nevertheless, the mechanisms linking PNI to metastasis and recurrence remain still unclear.

A century ago, PNI was identified as one of the routes of metastatic spread^4,5^. Several lines of evidence from recent studies have demonstrated nerve-cancer interaction^3,19–24^. Such evidence has given us a paradigm shift in the recognition of PNI. In short, this evidence indicates that an invaded nerve is not only a metastatic route but also a critical command center for the cancer stem cell niche during progression in PDAC.

Based on these findings, we can speculate that the nerve as a command center is able to make the tumor microenvironment (TME) favorable for pancreatic cancer itself. Accordingly, we hypothesized that the neural system modulated by nerve-cancer interaction, which resulted in PNI, also was associated with other changes (not the good kind) in the TME. However, there is little data about the relationship between PNI and other unfavorable changes in the TME in human PDAC specimens^14,25^. Therefore, this study aimed to evaluate resected pancreatic cancer tissues histologically and biologically and clarify what changes occurred alongside PNI. We present herein the relationships between PNI and venous and lymphatic invasion in PDAC. Furthermore, our proteomic analysis using resected human PDAC indicates that eukaryotic initiation factor 2 (EIF2) signaling, a critical pathway in response to integrated stress response (ISR), affects the severity of PNI.

## Results

### The invasion site of the extrapancreatic nerve plexus is different based on the location of PDAC

**Figure 1:**
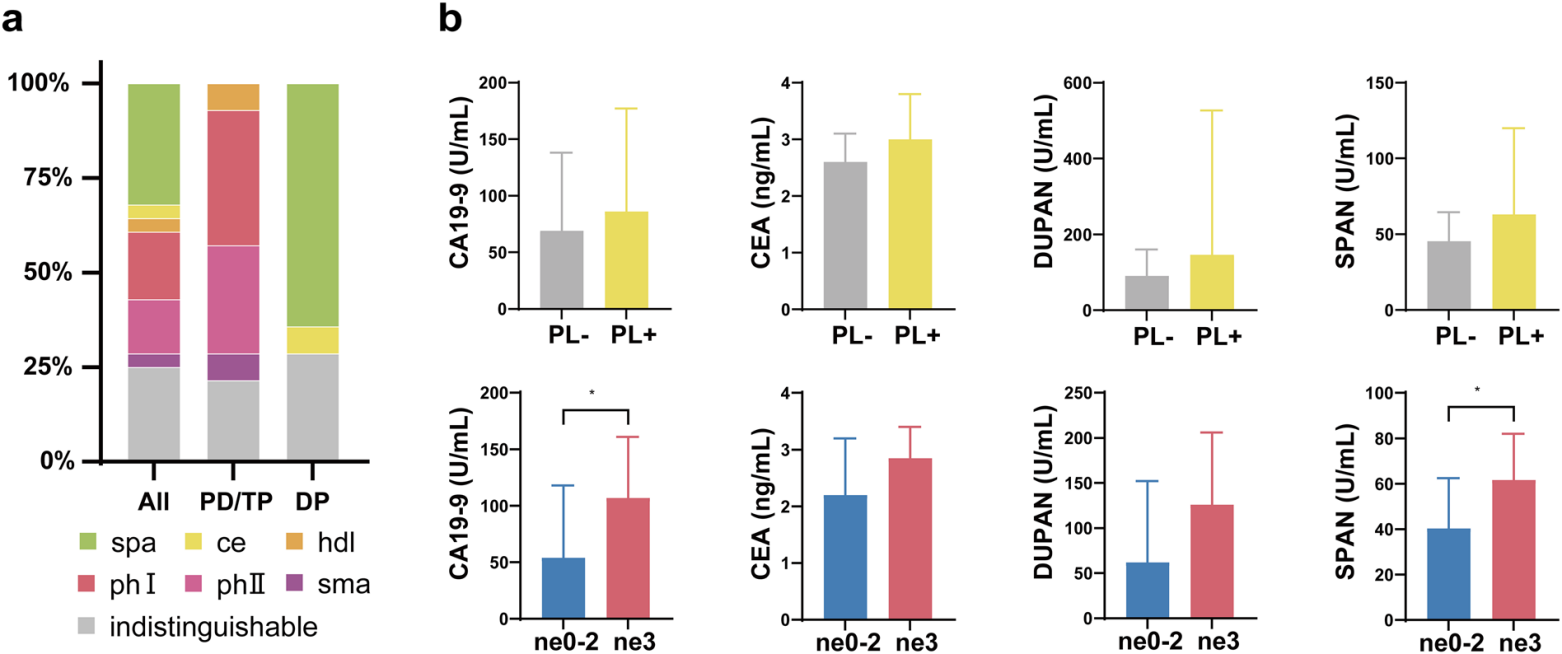
(**a**) The invasive sites of the extrapancreatic nerve plexus. *Ce* the celiac plexus, *DP* distal pancreatectomy, *hdl* the hepatoduodenal ligament nerve plexus, *PD* pancreatoduodenectomy, *phI* the pancreatic head nerve plexus I, *phII* the pancreatic head nerve plexus II, *sma* the superior mesenteric nerve plexus, *spa* the splenic artery nerve plexus, *TP* total pancreatectomy. (**b**) Comparison of tumor biomarkers. *CA19-9* carbohydrate antigen 19-9, *CEA* carcinoembryonic antigen, *DUPAN* duke pancreatic monoclonal antigen, *ne* nerve invasion, *PL* extrapancreatic nerve plexus invasion, *SPAN* s-pancreas antigen. “*” indicates significance at P < 0.05.

Of the 128 patients, 100 (78.1%) were included in the non-extrapancreatic nerve plexus invasion (PL) group and 28 patients (21.9%) were in the PL group. The invasive sites of the extrapancreatic nerve plexus are shown in Fig. 1a. The PL site with pancreatic head cancer mainly included the pancreatic head nerve plexuses I and II. In contrast, the PL site of pancreatic body and tail cancer were mainly in the splenic artery nerve plexus (64.3%).

### The high-grade ne group had higher tumor biomarker values, CA19-9 and SPAN, compared to the low-grade ne group

A comparison of the clinical characteristics and operation-related factors between the PL and non-PL groups is shown in Table 1 and Fig. 1b. There were no significant differences between the two groups according to tumor biomarker values.

**Table 1:** Comparison of preoperative, operation-related characteristics.

	All (n = 128)	Non-PL (n = 100)	PL (n = 28)	P value	Low-grade ne (n = 54)	High-grade ne (n = 74)	P value
Gender, male, n	65 (50.8)	53 (53.0)	12 (42.9)	0.343	21 (38.9)	44 (59.5)	0.022
Age, year	70 (50–85)	69 (52–85)	71 (50–82)	0.970	71 (52–82)	70 (50–85)	0.441
Body mass index, kg/m^2^	22.3 (14.1–36.3)	22.4 (15.8–36.3)	21.4 (14.1–33.3)	0.135	22.1 (16.2–33.3)	22.3 (14.1–36.3)	0.969
Obstructive jaundice, n	60 (46.9)	48 (48.0)	12 (42.9)	0.630	19 (35.2)	41 (55.4)	0.024
Diabetes mellitus, n	43 (33.6)	33 (33.0)	10 (35.7)	0.788	19 (35.2)	24 (32.4)	0.745
**Laboratory values**							
CA19-9, U/mL	71 (1–9675)	69 (1–9675)	86 (1–2378)	0.854	54 (1–2402)	107 (1–9675)	0.020
CEA, ng/mL	2.7 (0.5–37.0)	2.6 (0.5–37.0)	3.0 (0.5–10.5)	0.590	2.2 (0.5–37.0)	2.9 (0.5–23.9)	0.152
DUPAN, U/mL	96 (22–16,000)	91 (22–10,800)	146 (25–16,000)	0.167	62 (25–3676)	126 (22–16,000)	0.063
SPAN, U/mL	51 (2–2284)	45 (3–1667)	63 (2–2284)	0.666	40 (3–708)	61 (2–2284)	0.031
**Operative outcomes**							
Procedure, n				0.036			0.458
Pancreaticoduodenectomy	83 (64.8)	69 (69.0)	14 (50.0)		32 (59.3)	51 (68.9)	
Distal pancreatectomy	40 (31.3)	26 (26.0)	14 (50.0)		19 (35.2)	21 (28.4)	
Total pancreatectomy	5 (3.9)	5 (5.0)	0		3 (5.6)	2 (2.7)	
Operation time, min	310 (91–647)	317 (91–647)	283 (127–619)	0.334	278 (91–587)	317 (127–647)	0.185
Intraoperative blood loss, mL	765 (90–3915)	750 (90–3915)	783 (180–2450)	0.793	615 (150–2610)	955 (90–3915)	0.042
Intraoperative ABT, n	24 (18.8)	19 (19.0)	5 (17.9)	0.891	6 (11.1)	18 (24.3)	0.059
Portal vein resection, n	19 (14.8)	14 (14.0)	5 (17.9)	0.612	4 (7.4)	15 (20.3)	0.043

*ABT* allogeneic red blood cell transfusion, *CA19-9* carbohydrate antigen 19-9, *CEA* carcinoembryonic antigen, *DUPAN* duke pancreatic monoclonal antigen, *ne* nerve invasion, *PL* extrapancreatic nerve plexus invasion, *SPAN* s-pancreas antigen.

Any grade of nerve invasion was identified pathologically in the majority (123/128, 96%) of the resected pancreases for early-stage PDAC. The high-grade ne group included 74 cases (57.8%), and the low-grade ne group included 54 cases (42.2%). The high-grade ne groups showed a higher tumor biomarker value, such as CA19-9 (2-fold, P = 0.024) and SPAN (1.5-fold, P = 0.031) (Fig. 1b). This feature is not similar to the results attained comparing PL status. Greater intraoperative blood loss in the high-grade ne group (1940 vs. 650 mL, P < 0.001) was assumed to be associated with a higher frequency of preoperative obstructive jaundice and surgical resection combined with portal vein resection (Table 1).

### The PL group showed an increase in extrapancreatic local invasion compared to the non-PL group

The comparison of the pathological characteristics between the two groups is shown in Table 2. The maximum tumor size in the PL group was significantly larger than that of the non-PL groups. Moreover, the PL groups showed a higher prevalence of local invasion factors involving retropancreatic tissue, the portal venous system, the arterial system, and other organs.

**Table 2:** Comparison of pathological characteristics in the entire cohort.

	All (n = 128)	Non-PL (n = 100)	PL (n = 28)	P value	Low-grade ne (n = 54)	High-grade ne (n = 74)	P value
Tumor size, mm	30 (7–150)	30 (7–150)	35 (25–86)	0.014	26 (7–150)	35 (17–130)	< 0.001
**UICC 8th edition**							
**T category, n**				0.053			0.003
T1	16 (12.5)	16 (16.0)	0		13 (24.1)	3 (4.1)	
T2	79 (61.7)	61 (61.0)	18 (64.3)		30 (55.6)	49 (66.2)	
T3	33 (25.8)	23 (23.0)	10 (35.7)		11 (20.4)	22 (29.7)	
T4	0	0	0		0	0	
**N category, n**				0.852			0.002
N0	49 (38.3)	39 (39.0)	10 (35.7)		30 (55.6)	19 (25.7)	
N1	49 (38.3)	37 (37.0)	12 (42.9)		16 (29.6)	33 (44.6)	
N2	30 (23.4)	24 (24.0)	6 (21.4)		8 (14.8)	22 (29.7)	
**M category, n**				0.197			0.236
M0	117 (91.4)	93 (93.0)	24 (85.7)		51 (94.4)	66 (89.2)	
M1^a^	11 (8.6)	7 (7.0)	4 (14.3)		3 (5.6)	8 (10.8)	
**UICC Stage, n**				0.361			0.002
IA	12 (9.4)	12 (12.0)	0		11 (20.4)	1 (1.4)	
IB	24 (18.8)	18 (18.0)	6 (21.4)		13 (24.1)	11 (14.9)	
IIA	12 (9.4)	9 (9.0)	3 (10.7)		6 (11.1)	6 (8.1)	
IIB	45 (35.2)	34 (34.0)	11 (39.3)		14 (25.9)	31 (41.9)	
III	24 (18.8)	20 (20.0)	4 (14.3)		7 (13.0)	17 (23.0)	
IV	11 (8.6)	7 (7.0)	4 (14.3)		3 (5.6)	8 (10.8)	
**R0 resection, n**	114 (89.1)	92 (92.0)	22 (78.6)	0.054	49 (90.7)	65 (87.8)	0.603
**Local invasion factor, n**							
Bile duct invasion	60 (46.9)	49 (49.0)	11 (39.3)	0.363	18 (33.3)	42 (56.8)	0.009
Duodenal invasion	56 (43.8)	44 (44.0)	12 (42.9)	0.914	19 (35.2)	37 (50.0)	0.095
Serosal side of the anterior pancreatic tissue invasion	28 (21.9)	23 (23.0)	5 (17.9)	0.561	10 (18.5)	18 (24.3)	0.433
Retropancreatic tissue invasion	103 (80.5)	75 (75.0)	28 (100.0)	0.003	36 (66.7)	67 (90.5)	0.001
Portal venous system invasion	28 (21.9)	15 (15.0)	13 (46.4)	< 0.001	6 (11.1)	22 (29.7)	0.012
Arterial system invasion	20 (15.6)	11 (11.0)	9 (32.1)	0.010	2 (3.7)	18 (24.3)	0.002
Extrapancreatic nerve plexus invasion	28 (21.9)	–	–	–	5 (9.3)	23 (31.1)	0.003
Invasion of other organs	6 (4.7)	2 (2.0)	4 (14.3)	0.021	2 (3.8)	4 (5.4)	0.508
**Assessment of TME**							
**Lymphatic invasion, n**				0.672			0.001
No evidence of invasion	5 (3.9)	5 (5.0)	0		5 (9.3)	0	
Slight invasion	24 (18.8)	19 (19.0)	5 (17.9)		16 (29.6)	8 (10.8)	
Moderate invasion	59 (46.1)	45 (45.0)	14 (50.0)		19 (35.2)	40 (54.1)	
Marked invasion	40 (31.3)	31 (31.0)	9 (32.1)		14 (25.9)	26 (35.1)	
**Venous invasion, n**				0.022			< 0.001
No evidence of invasion	7 (5.5)	7 (7.0)	0		7 (13.0)	0	
Slight invasion	33 (25.8)	29 (29.0)	4 (14.4)		24 (44.4)	9 (12.2)	
Moderate invasion	58 (45.3)	46 (46.0)	12 (42.9)		17 (31.5)	41 (55.4)	
Marked invasion	30 (23.4)	18 (18.0)	12 (42.9)		6 (11.1)	24 (32.4)	
**Nerve invasion, n**				0.028			-
No evidence of invasion	5 (3.9)	5 (5.0)	0		–	–	
Slight invasion	15 (11.7)	13 (13.0)	2 (7.1)		–	–	
Moderate invasion	34 (26.6)	31 (31.0)	3 (10.7)		–	–	
Marked invasion	74 (57.8)	51 (51.0)	23 (82.1)		–	–	
**Cancer-stroma relationship, n**				0.569			0.026
Medullary type	4 (3.1)	4 (4.0)	0		3 (5.6)	1 (1.4)	
Intermediate type	74 (58.3)	58 (58.0)	16 (59.3)		37 (68.5)	37 (50.7)	
Scirrhous type	49 (38.6)	38 (38.0)	11 (40.7)		14 (25.9)	35 (47.9)	

*ne* nerve invasion, *PL* extrapancreatic nerve plexus invasion, *TME* tumor microenvironment, *UICC* Union for International Cancer Control.

^a^All of the patients were diagnosed with M1 due to positive lymph nodes other than the regional lymph nodes.

### The high-grade ne group showed an increase in local invasion and lymphatic metastasis compared to the low-grade ne group

There were significant differences in the pathological findings between the groups (Table 2). One of the critical features of the high-grade ne group was higher incidences of lymphatic metastasis (P = 0.002), with more advanced clinical stage. Furthermore, the prevalence of local invasion in the high-grade ne group was significantly higher than that of the low-grade ne groups. Similarly, the high-grade ne group was associated with much more invasiveness in the TME-related assessment.

### The high-grade ne group showed a higher incidence of early recurrence after curative surgery

There were no significant differences in the incidences of postoperative complications between the PL group and non-PL group (Table 3).

**Table 3:** Postoperative outcomes in the entire cohort.

	All (n = 128)	Non-PL (n = 100)	PL (n = 28)	P value	Low-grade ne (n = 54)	High-grade ne (n = 74)	P value
Postoperative complications (Clavien-dindo classification grade ≥ 3), n	24 (18.8)	20 (20.0)	4 (14.3)	0.494	10 (18.5)	14 (18.9)	0.954
Pancreatic fistula (ISGPF grade ≥ B), n	23 (18.0)	17 (17.0)	6 (21.4)	0.590	8 (14.8)	15 (20.3)	0.427
Delayed gastric emptying (ISGPS grade ≥ B), n	11 (8.6)	8 (8.0)	3 (10.7)	0.139	3 (5.6)	8 (6.3)	0.741
Postoperative hospital stay, day	19 (6–73)	20 (7–73)	17 (6–64)	0.322	19 (6–73)	19 (7–64)	0.862
Adjuvant chemotherapy, n	103 (83.1)	83 (83.0)	20 (74.1)	0.133	45 (84.9)	58 (81.7)	0.637
Recurrence within 6 month, n	36 (28.1)	27 (27.0)	9 (32.1)	0.593	8 (14.8)	28 (37.8)	0.004

*ISGPF* the International Study Group of Pancreatic Fistula, *ISGPS* the International Study Group of Pancreatic Surgery, *ne* nerve invasion, *PL* extrapancreatic nerve plexus invasion.

Similarly, there were no significant differences in the incidences of postoperative complications between the high-grade ne group and the low-grade ne group. However, patients with high-grade ne were linked to a higher incidence of early recurrence after surgery (37.8% vs. 14.8%, P = 0.004) (Table 3).

### PNI caused a poor prognosis after radical surgery for early-stage PDAC

**Figure 2:**
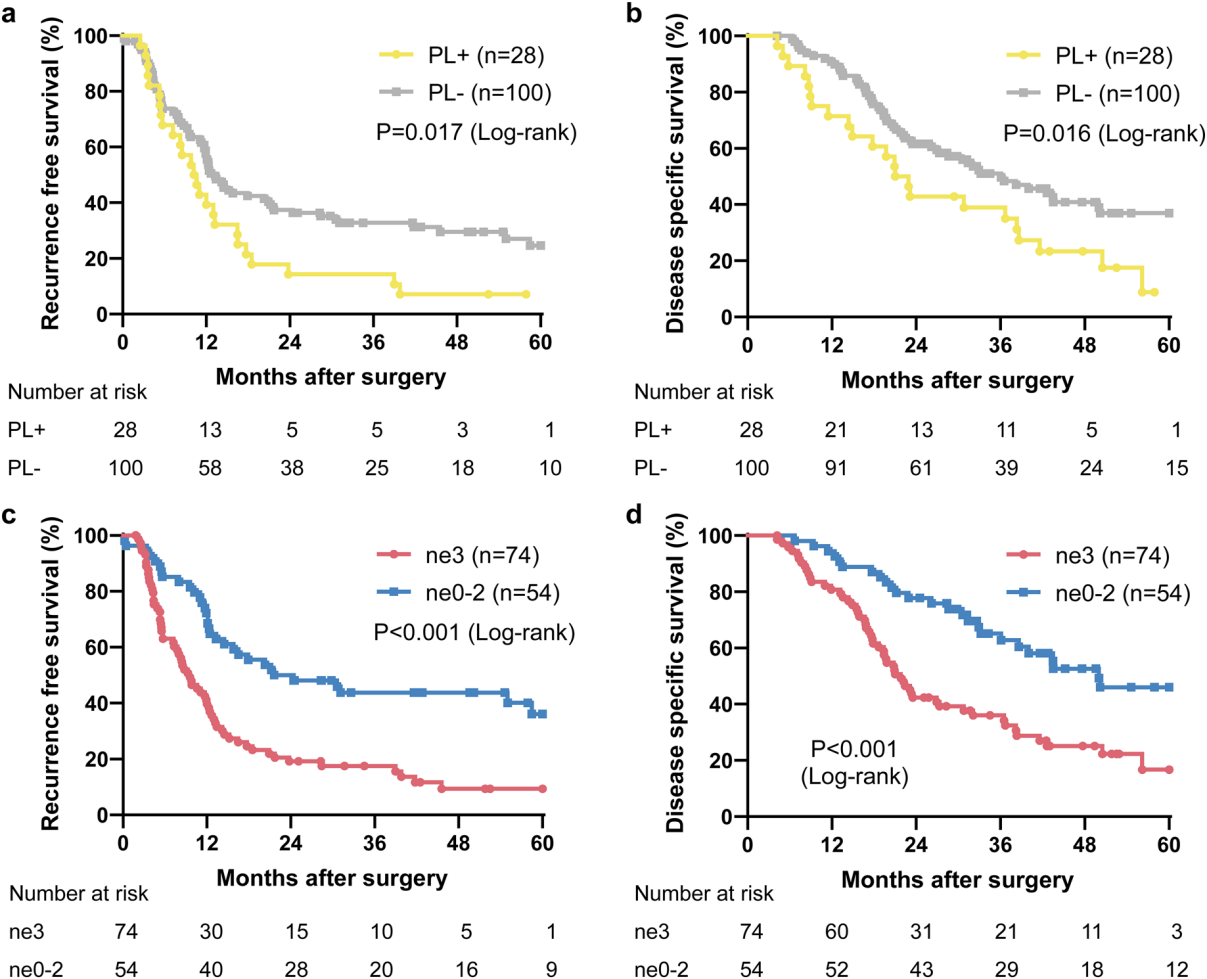
Survival analysis by log-rank test in the PL and non-PL groups, (**a**) Recurrence free survival (P = 0.017), (**b**) Disease specific survival (P = 0.016). Survival curves in the high-grade ne and low-grade ne groups, (**c**) Recurrence free survival (P < 0.001), (**d**) Disease specific survival (P < 0.001). *ne* nerve invasion, *PL* extrapancreatic nerve plexus invasion.

The recurrence free survival (RFS) and disease specific survival (DSS) curves for patients classified as PL are shown in Fig. 2. The RFS time was significantly shorter in the PL group than in the non-PL group (MST, 10.4 vs. 13.3 months, P = 0.017). The DSS was also significantly shorter in the PL group (MST, 22.0 vs. 36.1 months, P = 0.016). Likewise, The RFS time (MST, 9.6 vs. 23.0 months, P < 0.001) and the DSS time (MST, 21.8 vs. 50.0 months, P < 0.001) were significantly shorter in the high-grade ne group than in the low-grade ne group.

### The lymphatic and venous invasions occurred alongside nerve invasion

We further characterized the relationship between ne and other TME-related features. In the current study, we evaluated lymphatic invasion and venous invasion as TME-related features. Moreover, we calculated the polychoric correlation coefficient. There were only a few patients with no invasion of the lymphatic and venous systems even in the early-stage PDAC.

**Figure 3:**
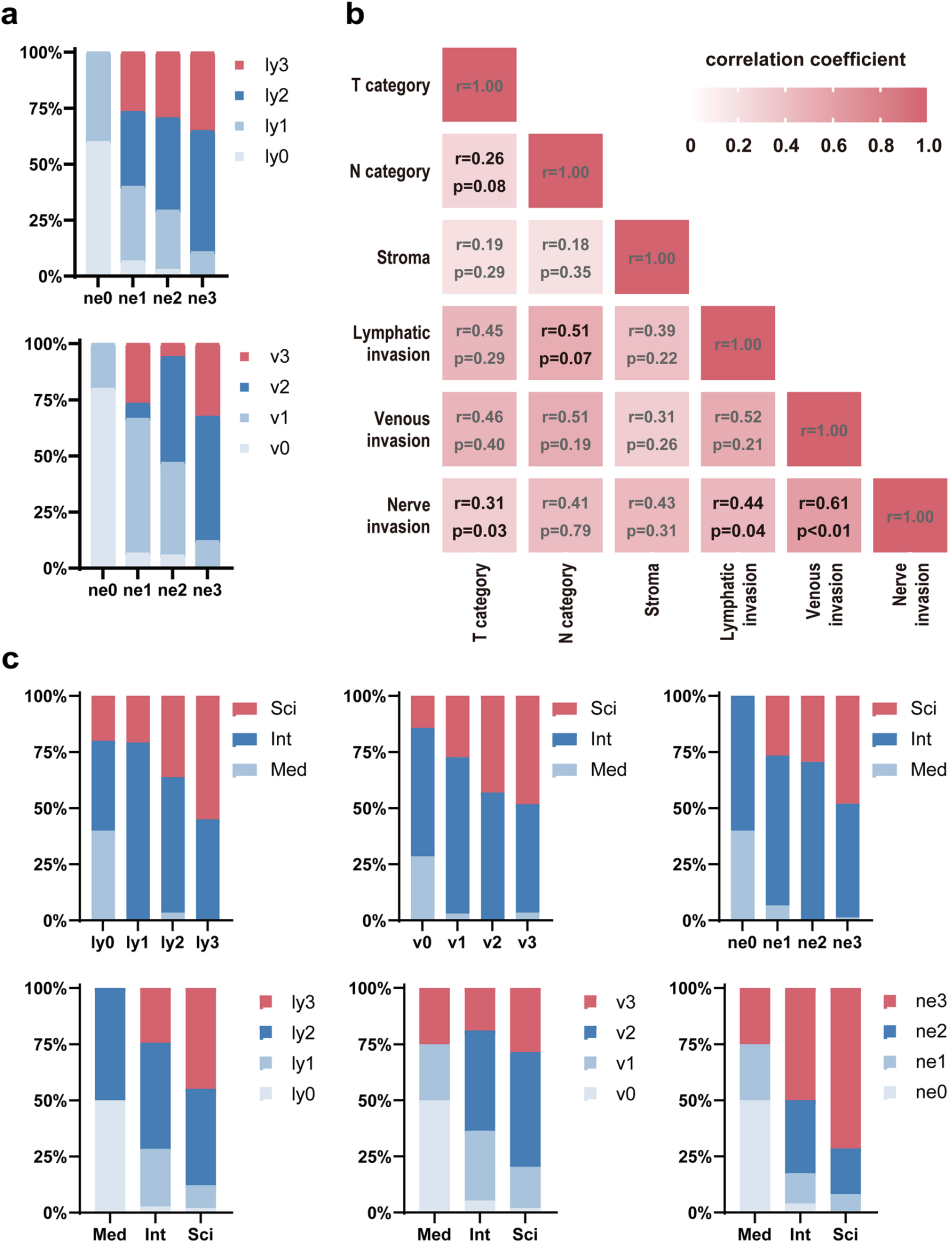
The relationship between nerve invasion and other pathological findings. (**a**) The lymphatic and venous invasions occurred alongside nerve invasion even in the early-stage PDAC. (**b**) The polychoric correlations among the pathological findings. (**c**) The correlation between the density of the stromal component and lymphatic invasion, nerve invasion, and venous invasion. *Int* intermediate type, *ly* lymphatic invasion, *med* medullary type, *ne* nerve invasion, *sci* scirrhous type, *v* venous invasion.

In the cases without nerve invasion, there were no cases with moderate or more invasion of lymphatic and venous systems. In contrast, it was revealed that around 90% of the cases with marked nerve invasion had moderate or more severe invasion of the lymphatic and venous systems (Fig. 3a). We found a significant positive correlation between nerve invasion and lymphatic invasion (r = 0.440, P = 0.042), as well as nerve invasion and venous invasion (r = 0.610, P = 0.002), respectively (Fig. 3b). Unexpectedly, there was no correlation between lymphatic invasion and venous invasion. Collectively, these observations suggest that regulating factors that cause nerve invasion may also control venous and lymphatic invasion in the TME of PDAC.

### Tumors having abundant stroma often displayed lymphatic, venous, and nerve invasions

To investigate the regulating factors that affect nerve, lymphatic and venous invasion, we evaluated the stroma, which is a main component of the TME. Because it has been reported that stroma cells produce neural-related factors that facilitate tumor cell proliferation^21^, we focused on the stroma. Seventy-four of the 128 patients (58.3%) had the intermediate type, and 49 patients (38.6%) had the scirrhous type tumor containing abundant stroma. Even in resectable early-stage PDAC cases, the medullary type (scant stroma) was rare (Table 2). Although a statistically significant difference could not be clearly determined due to the small number of medullary cases, the density of stromal composition seemed to be a factor involved in patient survival (Supplementary Fig. S1).

Next, we performed a factor analysis among the stroma and other TME-related factors using a polychoric correlation. We found that a dense stromal component tended to be correlated with lymphatic invasion (r = 0.390, P = 0.222), nerve invasion (r = 0.429, P = 0.311), and venous invasion (r = 0.310, P = 0.259), respectively (Fig. 3b,c). These data suggest that cancer invasiveness into the peripancreatic structures can be promoted by cancer-stroma interaction in a dose-dependent manner.

### Proteomic profiling of the high-grade ne group compared with the low-grade ne group

To gain insights into the characterization of the high-grade ne group compared to the low-grade ne group, comprehensively, we analyzed the resected pancreas using a label-free LC–MS/MS proteomics analysis. To clearly uncover distinctive differences between the groups, we excluded the ne2 cases from the target in proteomics analysis. Additionally, to minimize the influence by the tumor location on the proteomic results, we only analyzed the cancers of the head and neck of the pancreas for which a pancreaticoduodenectomy was performed. Finally, eight randomly chosen resected pancreases were examined for each group.

We used 1054 quantified proteins to determine the significance of differences in protein expression by a q-value cutoff set at < 0.1 as the threshold. Next, we performed two group comparison to find the discriminating variables between the low-grade ne group and the high-grade ne group. One hundred two of 1054 proteins (9.8%) were identified as significant differentially expressed proteins after statistical analysis (P < 0.05). Among them, 39 proteins (38.2%) were up-regulated and 63 proteins (61.8%) were down-regulated in the high-grade ne group.

### Eukaryotic initiation factor-2 signaling was the most significantly enriched pathway in the high-grade ne group

**Figure 4:**
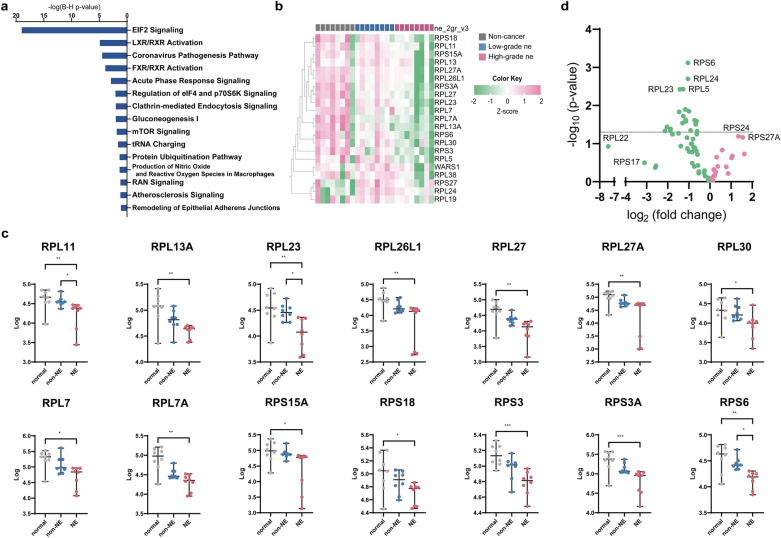
(**a**) The core analysis of top canonical pathways. (**b**) The heatmap of significantly differentially expressed proteins related to EIF2 signaling. This was generated with Qlucore Omics Explorer v3.7 (https://qlucore.com). (**c**) The comparison of the intensity of molecules involved in EIF2 signaling. “*”, “**”, and “***” indicate significance at P < 0.05, P < 0.01 and P < 0.001, respectively. *non-NE* low-grade ne, *NE* high-grade ne. (**d**) The volcano plot of the identified ribosomal proteins in our dataset. Red circles show the increased expression in the high-grade ne group than in the low-grade ne group. Green circles show the decreased expression in the high-grade ne group than in the low-grade ne group.

To systematically explore the pathway that changes in the high-grade ne group, a dataset that included all the identified differentially expressed proteins against the low-grade ne group was submitted to QIAGEN IPA for canonical pathway analysis. The differentially expressed proteins were categorized as to related canonical pathways based on the Ingenuity pathway knowledge base. The top enriched categories of canonical pathways with a p-value cutoff set at < 0.05 by Benjamini–Hochberg correction [a-log (B–H p-value) greater than 1.5] are shown in Fig. 4a.

In the high-grade ne group, the most significantly enriched pathway was EIF2 signaling [z-score: − 3.5, p-value = 3.10E−22, Ratio: 21/224 (0.094)]. In the high-grade ne group, the most significantly activated pathway was LXR/RXR activation (z-score: 2.1). Interestingly, in addition to EIF2 signaling, enriched categories of canonical pathways included various pathways involved in protein synthesis and degradation such as regulation of eIF4 and p70S6K signaling, mTOR signaling, the protein ubiquitination pathway, and the unfolded protein response. Collectively, these data indicate the regulation of protein homeostasis (proteostasis) in the case of high-grade ne can be different from that of low-grade ne.

### Ribosomal proteins, which are subunits of EIF2 signaling, showed decreased expression according to the severity of ne

To clarify whether inactivation of EIF2 signaling is a characteristic finding in the cancerous part, we next compared the proteomic data of the non-cancerous part of the resected specimen between the high-grade ne group and the low-grade ne group. We created a heatmap of the differentially expressed proteins which are related to EIF2 signaling. We used a multi-group comparison with the Kruskal–Wallis test followed Dunn’s correction. In contrast to the non-cancerous part, the cancerous part demonstrated significant decrease in the expression of various ribosomal proteins which is a subunit of EIF2 signaling. Furthermore, it is likely down-regulated according to the severity of ne (Fig. 4b,c).

Next, to perform further analysis of specialized ribosomal proteins, we compared the discriminating proteins between the low-grade ne group and the high-grade ne group to the ribosomal protein list taken from the IPA knowledge base, and then identified the ribosomal proteins that had increased or decreased in our dataset. As a result, seventy ribosomal proteins were identified. Figure 4d shows a volcano plot based on the identified ribosomal proteins. Many ribosomal proteins that have been identified in our dataset showed the decreased expression, but not all. These results suggest that there is a difference in ribosome biogenesis and function between low-grade ne PDAC and high-grade ne PDAC.

## Discussion

This study revealed that PNI was strongly associated with poor prognosis in patients who underwent resection with curative intent for early-stage PDAC. In addition, this study also demonstrated that lymphatic and venous invasions happened alongside the nerve invasion with a positive correlation in their severity. Previous reports showed that PNI was encountered in nearly 100% of resected PDAC specimens^11,14,26,27^. Our study was also consistent with that. In other words, surprisingly, we found PNI in almost all patients with early-stage PDAC. Furthermore, in contrast to previous reports^14,25^, our results demonstrated a positive correlation between other TME-related features and PNI. These results evoked the notion that some cues which contribute to PNI make the TME favorable to the cancer itself and thus promote cancer progression and metastasis.

What are the cues? One candidate is the stroma. We found a positive correlation between cancer aggressiveness, such as PNI, and a dense stroma. Ceyhan et al. also suggested that desmoplasia may be a factor triggering increased cancer cell invasiveness and thus PNI^27^. One of the most distinctive morphological features of PDAC is its dense desmoplastic stroma^28–30^. The stroma, composed of a mixture of extracellular matrix and non neoplastic cells, has harmful effects^29,31^. The stroma consists of proliferating fibroblasts and pancreatic stellate cells that produce and deposit fibronectin and collagens, inflammatory cells that produce chemokines and cytokines, and nerve fibers that release nerve growth factors^32–34^. Recently, PDAC data show that activated stellate cells support PNI in PDAC^27,35^. Activated stellate cells are also a pivotal component of the desmoplastic reaction that correlates with neuropathic changes in PDAC^36–38^.

Stroma cells produce neural-related factors, which leads to tumor cell proliferation and survival in the primary site and secondary site^21^. Neural cells and neural-related factors have been increasingly perceived as major modulators of the aggressiveness of this lethal disease^19,26,39–41^. PNI is regulated by the interaction between the nerve microenvironment and neurotrophic molecules from cancer cells^11,21,42^. Several studies have demonstrated that high expression of nerve growth factor (NGF) and its receptor tropomyosin related kinase A (TrkA) correlated with the presence of PNI in cancer^43,44^. Ma et al. showed the presence of NGF and TrkA expression in human pancreatic cancer by immunohistochemistry (IHC) and reverse transcription polymerase chain reaction. The positive rate of NGF by IHC was 82.4%. Interestingly, NGF expression was correlated with a more frequent presence of PNI as well as lymph node metastasis^43^. Our clinical data also demonstrated that high-grade ne is accompanied by a higher grade of lymphatic and vascular invasion and a higher incidence of lymph node metastasis than low-grade ne. Taken together, these data suggest that NGF and its receptors can cause PNI and make the TME favorable to cancer progression and metastasis.

The notable feature of the high-grade ne group on proteomic analysis is the inactivation of EIF2 signaling. EIF2 signaling significantly plays an essential role in the ISR to maintain proteostasis. Protein kinases that phosphorylate the alpha subunit of EIF2 are activated in stressed cells and negatively regulate protein synthesis^45–49^. The dysregulated ISR signaling and loss of proteostasis are associated with the pathogenesis of various diseases, including cognitive disorders, diabetes, metabolic disorders, neurodegeneration, and cancer^45^. ISR signaling is activated in response to exposure to various environmental stress such as hypoxia and nutrient deprivation^46,50,51^. Such stressors are typical hallmarks in the TME of PDAC, which contribute to cancer progression. Furthermore, a dense stroma enhances the stress^33,52–55^. Based on these pieces of evidence, the high-grade ne group with abundant stroma can be thought to have been under much more environmental stress compared to the low-grade ne group, lacking stroma.

In order to resist cancer progression, high-grade ne cases must need to activate ISR signaling via EIF2 signaling. Nevertheless, in our proteomic analysis, EIF2 signaling was inactivate in the high-grade ne group. Additionally, we found differences in various canonical pathways involved in proteostasis between the low-grade and the high-grade ne groups. Collectively, our data indicate that there is an underlying difference in proteostasis between the groups and that leads to the difference in their prognosis.

Our study demonstrated novel insight into underlying factors in high-grade ne PDAC, however, there are several limitations. Though we found a difference regarding EIF2 signaling and ribosome biogenesis, we have not yet clarified the translational activity of ribosomes and the detailed molecular mechanism of how those pathways are connected to PNI in PDAC. Both proteostasis and ribosome biogenesis are associated with cancer and have the potential of being therapeutic targets^56–58^. However, EIF2 signaling, in particular, has a paradox due to the complexity of the regulation, in that it controls both pro-survival and pro-death mechanisms^45^. A better understanding of the underlying mechanism is necessary for this promising candidate to take its position in clinical settings.

In conclusion, the present study showed PNI is linked with lymphatic and vascular invasion in early-stage PDAC. The severity of PNI is associated with abundant stroma. The dysregulation of proteostasis and ribosome biogenesis can yield a difference in the severity of PNI.

## Methods

### Patients

This single-center, retrospective, observational study was approved by the Committee of Medical Ethics of Hirosaki University Graduate School of Medicine (reference no. 2020-203). Informed consent was obtained in the form of opt-out on our website (https://www.med.hirosaki-u.ac.jp/hospital/outline/resarch/resarch.html), with the approval of the Committee of Medical Ethics of Hirosaki University Graduate School of Medicine. This study was designed and carried out in accordance with the Declaration of Helsinki.

Patients at our facility undergoing pancreatic surgery, with curative intent, for early-stage resectable PDAC, between January 2007 and May 2018, were considered for this study. Of those, the 128 patients who did not meet any of the exclusion criteria (Supplementary Content 1) were analyzed. None of the included patients received neoadjuvant therapy before surgery. Resectability status was made based on National Comprehensive Cancer Network guidelines.

### Surgical procedures and operative management

Supplementary Content 2.

### Histological grading of pancreatic cancers

All slides that were originally prepared from formalin-fixed and paraffin-embedded tissue were reviewed. Morphological analyses were performed using slides stained with hematoxylin and eosin. Grading of histological findings of the resected pancreatic tissues was performed referring to an already published scoring system for pancreatic cancer^59^. H&E stained pancreatic sections were graded on three criteria: nerve invasion (ne), venous invasion (v), and lymphatic invasion (ly), on the following scales. They were graded as 0 = no evidence of invasion, 1 = slight invasion, 2 = moderate invasion, and 3 = marked invasion, based on previous reports^60–62^. In this grading system, ne/v/ly are associated with pathological specimens prepared from the section of tumor at the “largest tumor diameter”. Slight invasion means 1 or 2 foci of invasion; moderate invasion is 3 or 4 foci; and marked invasion is 5 ≦ foci. We also evaluated the local invasion factors based on this scoring system^59^. Extrapancreatic nerve plexus invasion (PL) was assessed as absent or present. We further evaluated the cancer-stroma relationship^59^. Tumors were classified into the following types according to the proportion of stroma they contained: medullary type (med), tumors containing scant stroma; intermediate type (int), tumors containing a proportion of stroma intermediate between the scirrhous type and the medullary type; scirrhous type (sci), or tumors containing abundant stroma. The slides were examined by board-certified pathologists unaware of the clinical data.

### Comparison of perioperative factors

The 128 patients were divided into two groups according to their pathological states; absence of PL invasion was defined as the non-PL group, and a grade of nerve invasion score less than 3 was defined as the low-grade ne group. The medical records for each case were reviewed and compared between the two groups.

### Liquid chromatography with tandem mass spectrometry (LC–MS/MS)

Supplementary Content 3.

### Proteomics data analysis

Supplementary Content 4.

### Other statistical analyses

Supplemental Content 5.

### Ethics approval and consent to participate

This study was approved by the Committee of Medical Ethics of Hirosaki University Graduate School of Medicine (reference no. 2020-203). Informed consent was obtained in the form of opt-out on our website (https://www.med.hirosaki-u.ac.jp/hospital/outline/resarch/resarch.html), with the approval of the Committee of Medical Ethics of Hirosaki University Graduate School of Medicine. This study was designed and carried out in accordance with the Declaration of Helsinki.

### Consent for publication

Informed consent was obtained in the form of opt-out on our website (https://www.med.hirosaki-u.ac.jp/hospital/outline/resarch/resarch.html), with the approval of the Committee of Medical Ethics of Hirosaki University Graduate School of Medicine.

## Supplementary Information

Supplementary Information 1.

Supplementary Information 2.

Supplementary Information 3.

Supplementary Information 4.

Supplementary Information 5.

Supplementary Information 6.

Supplementary Information 7.

## Data Availability

The proteomic datasets generated and/or analysed during the current study are available online using access number “PXD025975’’ for the Proteome Xchange site^63^ and access number “JPST001172’’ for the jPOST Repository^64^.

## References

[1] Bray F (2018). Global cancer statistics 2018: GLOBOCAN estimates of incidence and mortality worldwide for 36 cancers in 185 countries. CA Cancer J. Clin..

[2] Siegel RL, Miller KD, Jemal A (2020). Cancer statistics, 2020. CA Cancer J. Clin..

[3] Amit M, Na'ara S, Gil Z (2016). Mechanisms of cancer dissemination along nerves. Nat. Rev. Cancer.

[4] Demir IE (2010). Neural invasion in pancreatic cancer: The past, present and future. Cancers.

[5] Liebig C, Ayala G, Wilks JA, Berger DH, Albo D (2009). Perineural invasion in cancer: A review of the literature. Cancer.

[6] Jurcak NR (2019). Axon guidance molecules promote perineural invasion and metastasis of orthotopic pancreatic tumors in mice. Gastroenterology.

[7] Dwivedi S, Krishnan A (2020). Neural invasion: A scenic trail for the nervous tumor and hidden therapeutic opportunity. Am. J. Cancer Res..

[8] Liebl F (2014). The impact of neural invasion severity in gastrointestinal malignancies: A clinicopathological study. Ann. Surg..

[9] Cartwright T, Richards DA, Boehm KA (2008). Cancer of the pancreas: Are we making progress? A review of studies in the US Oncology Research Network. Cancer Control J. Moffitt Cancer Cent..

[10] Lowenfels AB, Maisonneuve P (2006). Epidemiology and risk factors for pancreatic cancer. Best Pract. Res. Clin. Gastroenterol..

[11] Bapat AA, Hostetter G, Von Hoff DD, Han H (2011). Perineural invasion and associated pain in pancreatic cancer. Nat. Rev. Cancer.

[12] Alrawashdeh W (2019). Perineural invasion in pancreatic cancer: Proteomic analysis and in vitro modelling. Mol. Oncol..

[13] Makino I (2008). Nerve plexus invasion in pancreatic cancer: Spread patterns on histopathologic and embryological analyses. Pancreas.

[14] Nakao A, Harada A, Nonami T, Kaneko T, Takagi H (1996). Clinical significance of carcinoma invasion of the extrapancreatic nerve plexus in pancreatic cancer. Pancreas.

[15] Schorn S (2017). The influence of neural invasion on survival and tumor recurrence in pancreatic ductal adenocarcinoma—A systematic review and meta-analysis. Surg. Oncol..

[16] Ozaki H (1999). The prognostic significance of lymph node metastasis and intrapancreatic perineural invasion in pancreatic cancer after curative resection. Surg. Today.

[17] Takahashi H (2012). Perineural invasion and lymph node involvement as indicators of surgical outcome and pattern of recurrence in the setting of preoperative gemcitabine-based chemoradiation therapy for resectable pancreatic cancer. Ann. Surg..

[18] Lu M (2019). Extrapancreatic neuropathy correlates with early liver metastasis in pancreatic head adenocarcinoma. Onco. Targets Ther..

[19] Zahalka AH, Frenette PS (2020). Nerves in cancer. Nat. Rev. Cancer.

[20] Griffin N, Faulkner S, Jobling P, Hondermarck H (2018). Targeting neurotrophin signaling in cancer: The renaissance. Pharmacol. Res..

[21] Kuol N, Stojanovska L, Apostolopoulos V, Nurgali K (2018). Role of the nervous system in cancer metastasis. J. Exp. Clin. Cancer Res. CR.

[22] Demir IE, Friess H, Ceyhan GO (2015). Neural plasticity in pancreatitis and pancreatic cancer. Nat. Rev. Gastroenterol. Hepatol..

[23] Faulkner S, Jobling P, March B, Jiang CC, Hondermarck H (2019). Tumor Neurobiology and the war of nerves in cancer. Cancer Discov..

[24] Jobling P (2015). Nerve-cancer cell cross-talk: A novel promoter of tumor progression. Cancer Res..

[25] Nagakawa T (1992). A clinicopathologic study on neural invasion in cancer of the pancreatic head. Cancer.

[26] Demir IE, Friess H, Ceyhan GO (2012). Nerve-cancer interactions in the stromal biology of pancreatic cancer. Front. Physiol..

[27] Ceyhan GO (2009). Pancreatic neuropathy and neuropathic pain—A comprehensive pathomorphological study of 546 cases. Gastroenterology.

[28] Hruban RH (2019). Why is pancreatic cancer so deadly? The pathologist's view. J. Pathol..

[29] Feig C (2012). The pancreas cancer microenvironment. Clin. Cancer Res..

[30] Neesse A (2019). Stromal biology and therapy in pancreatic cancer: Ready for clinical translation?. Gut.

[31] Whatcott CJ, Han H, Von Hoff DD (2015). Orchestrating the tumor microenvironment to improve survival for patients with pancreatic cancer: Normalization, not destruction. Cancer J. (Sudbury, Mass.).

[32] Korc M (2007). Pancreatic cancer-associated stroma production. Am. J. Surg..

[33] Hakamada K (2019). Cancer stroma-targeting therapy: A new tool for fighting pancreatic cancer?. Ann. Gastroenterol. Surg..

[34] Hosein AN, Brekken RA, Maitra A (2020). Pancreatic cancer stroma: An update on therapeutic targeting strategies. Nat. Rev. Gastroenterol. Hepatol..

[35] Li X (2014). Sonic hedgehog paracrine signaling activates stromal cells to promote perineural invasion in pancreatic cancer. Clin. Cancer Res..

[36] Apte MV (2004). Desmoplastic reaction in pancreatic cancer: Role of pancreatic stellate cells. Pancreas.

[37] Vonlaufen A (2008). Pancreatic stellate cells: Partners in crime with pancreatic cancer cells. Cancer Res..

[38] Samkharadze T (2011). Pigment epithelium-derived factor associates with neuropathy and fibrosis in pancreatic cancer. Am. J. Gastroenterol..

[39] Renz BW (2018). β2 adrenergic-neurotrophin feedforward loop promotes pancreatic cancer. Cancer Cell.

[40] Entschladen F, Palm D, Niggemann B, Zaenker KS (2008). The cancer's nervous tooth: Considering the neuronal crosstalk within tumors. Semin. Cancer Biol..

[41] Wakiya T, Ishido K, Yoshizawa T, Kanda T, Hakamada K (2021). Roles of the nervous system in pancreatic cancer. Ann. Gastroenterol. Surg..

[42] He S (2014). GFRα1 released by nerves enhances cancer cell perineural invasion through GDNF-RET signaling. Proc. Natl. Acad. Sci. U. S. A..

[43] Ma J, Jiang Y, Jiang Y, Sun Y, Zhao X (2008). Expression of nerve growth factor and tyrosine kinase receptor A and correlation with perineural invasion in pancreatic cancer. J. Gastroenterol. Hepatol..

[44] Kolokythas A, Cox DP, Dekker N, Schmidt BL (2010). Nerve growth factor and tyrosine kinase A receptor in oral squamous cell carcinoma: Is there an association with perineural invasion?. J. Oral Maxillofac. Surg..

[45] Costa-Mattioli M, Walter P (2020). The integrated stress response: From mechanism to disease. Science.

[46] Harding HP (2003). An integrated stress response regulates amino acid metabolism and resistance to oxidative stress. Mol. Cell.

[47] Kroemer G, Mariño G, Levine B (2010). Autophagy and the integrated stress response. Mol. Cell.

[48] Humeau J (2020). Phosphorylation of eukaryotic initiation factor-2α (eIF2α) in autophagy. Cell Death Dis..

[49] Harding HP (2000). Regulated translation initiation controls stress-induced gene expression in mammalian cells. Mol. Cell.

[50] Palam LR, Gore J, Craven KE, Wilson JL, Korc M (2015). Integrated stress response is critical for gemcitabine resistance in pancreatic ductal adenocarcinoma. Cell Death Dis..

[51] Pakos-Zebrucka K (2016). The integrated stress response. EMBO Rep..

[52] Onodera T (2020). Human pancreatic cancer cells under nutrient deprivation are vulnerable to redox system inhibition. J. Biol. Chem..

[53] Kamphorst JJ (2015). Human pancreatic cancer tumors are nutrient poor and tumor cells actively scavenge extracellular protein. Cancer Res..

[54] Shah VM, Sheppard BC, Sears RC, Alani AW (2020). Hypoxia: Friend or foe for drug delivery in pancreatic cancer. Cancer Lett..

[55] Erkan M (2012). The role of stroma in pancreatic cancer: Diagnostic and therapeutic implications. Nat. Rev. Gastroenterol. Hepatol..

[56] Pelletier J, Thomas G, Volarević S (2018). Ribosome biogenesis in cancer: New players and therapeutic avenues. Nat. Rev. Cancer.

[57] Derenzini M, Montanaro L, Trerè D (2017). Ribosome biogenesis and cancer. Acta Histochem..

[58] Bustelo XR, Dosil M (2018). Ribosome biogenesis and cancer: Basic and translational challenges. Curr. Opin. Genet. Dev..

[59] *Classification of Pancreatic Carcinoma*. 4th English ed. (Kanehara & Co., Ltd., 2017).

[60] Japanese Society for Cancer of the Colon and Rectum (2019). Japanese Classification of Colorectal, Appendiceal, and Anal Carcinoma: the 3d English Edition [Secondary Publication]. J. Anus Rectum Colon.

[61] Miyazaki M (2015). Classification of biliary tract cancers established by the Japanese Society of Hepato-Biliary-Pancreatic Surgery: 3(rd) English edition. J. Hepatobiliary Pancreat. Sci..

[62] Kumar V, Abbas AK, Aster JC (2021). Robbins and Cotran pathologic basis of disease.

[63] Deutsch EW (2017). The ProteomeXchange consortium in 2017: Supporting the cultural change in proteomics public data deposition. Nucleic Acids Res..

[64] Okuda S (2017). jPOSTrepo: An international standard data repository for proteomes. Nucleic Acids Res..

