# Supplementary material for: Eukaryotic initiation factor 2 signaling behind neural invasion linked with lymphatic and vascular invasion in pancreatic cancer
Source: Sci Rep. 2021 Oct 27;11:21197. doi: 10.1038/s41598-021-00727-3 (PMC8551178; doi:10.1038/s41598-021-00727-3)
Supplement: Supplementary file 1 — Supplementary Information 1. [file 41598_2021_727_MOESM1_ESM.docx]

**Figure legend for Supplementary Figure S1**

Survival analysis based on the density of stromal composition. a: Recurrence free survival (P = 0.085), b: Disease specific survival (P = 0.114).

Int, intermediate type; Med, medullary type; Sci, scirrhous type
