# Supplementary figures and images for: Eukaryotic initiation factor 2 signaling behind neural invasion linked with lymphatic and vascular invasion in pancreatic cancer
Source: Sci Rep. 2021 Oct 27;11:21197. doi: 10.1038/s41598-021-00727-3 (PMC8551178; doi:10.1038/s41598-021-00727-3)

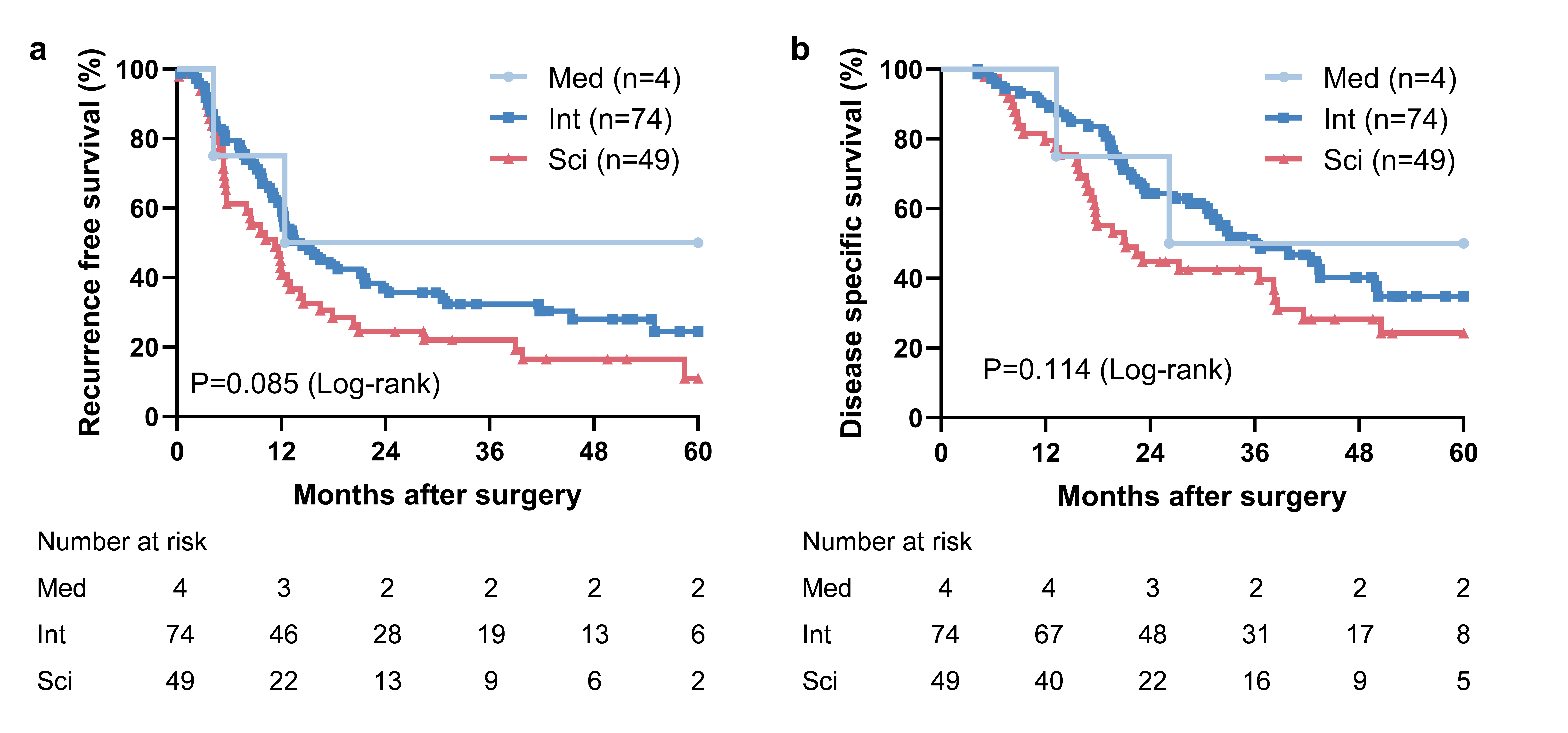

Supplement: Supplementary file 2 — Supplementary Information 2. [file 41598_2021_727_MOESM2_ESM.tif]
