# Supplementary material for: Eukaryotic initiation factor 2 signaling behind neural invasion linked with lymphatic and vascular invasion in pancreatic cancer
Source: Sci Rep. 2021 Oct 27;11:21197. doi: 10.1038/s41598-021-00727-3 (PMC8551178; doi:10.1038/s41598-021-00727-3)
Supplement: Supplementary file 3 — Supplementary Information 3. [file 41598_2021_727_MOESM3_ESM.docx]

**Supplemental Content 1**

***Patients***

The exclusion criteria were as follows: received neoadjuvant chemotherapy (n = 8), remnant pancreatic cancer (n = 3), other synchronous malignancies (n = 2), and missing data on follow-up (N = 1).
