# Supplementary material for: Eukaryotic initiation factor 2 signaling behind neural invasion linked with lymphatic and vascular invasion in pancreatic cancer
Source: Sci Rep. 2021 Oct 27;11:21197. doi: 10.1038/s41598-021-00727-3 (PMC8551178; doi:10.1038/s41598-021-00727-3)
Supplement: Supplementary file 4 — Supplementary Information 4. [file 41598_2021_727_MOESM4_ESM.docx]

**Supplemental Content 2**

***Surgical procedures and operative management***

We selected the type of pancreatic resection based on the tumor location. Open pancreatoduodenectomy with lymph node dissections was usually performed for cases of pancreatic head cancer. In cases of pancreatic body and tail cancer, open or minimally invasive distal pancreatectomy was performed with lymph node dissections. If swelling of a paraaortic lymph node was detected, we generally performed paraaortic lymph node sampling during the pancreatoduodenectomy, whereas sampling was not routinely performed during distal pancreatectomy. We performed a fresh frozen section analysis to confirm if the pancreatic cut-end margin was clear of residual cancer. If residual cancer was present at the pancreatic cut-end margin, we cut the pancreas further to reach negative margin status. If necessary, to achieve curative resection, we performed a total pancreatectomy with lymph node dissections.
