# Supplementary material for: Eukaryotic initiation factor 2 signaling behind neural invasion linked with lymphatic and vascular invasion in pancreatic cancer
Source: Sci Rep. 2021 Oct 27;11:21197. doi: 10.1038/s41598-021-00727-3 (PMC8551178; doi:10.1038/s41598-021-00727-3)
Supplement: Supplementary file 5 — Supplementary Information 5. [file 41598_2021_727_MOESM5_ESM.docx]

**Supplemental Content 3**

***Liquid Chromatography with Tandem Mass Spectrometry (LC-MS/MS)***

Formalin-fixed and paraffin-embedded pancreas tissues were deparaffinized by two changes of xylene and washed with a descending concentrations of ethanol. Proteins in the deparaffinized tissues were extracted in 20% (w/v) of 200 mM Tris-HCl (pH 8.8) containing 2% SDS and 0.2 M DTT followed by incubation at 100˚C for 20 min and shaking incubation at 80˚C for 2 h. The extracted proteins were precipitated by adding acetone. The precipitated proteins were resuspended in 10 μl of 500 mM ammonium bicarbonate and denatured with an equivalent volume of trifluoroethanol. Free cysteine residues were alkylated with 4 μl of 200 mM iodoacetamide for 60 min at room temperature in the dark and the remaining iodoacetamide was quenched by adding 1 μl of 200 mM DTT. The samples were then mixed with 300 μl of 100 mM ammonium bicarbonate. Fifteen microliters of the sample was diluted with 85 μl of 100 mM ammonium bicarbonate and incubated with 1 μg trypsin (TPCK treated, AB Sciex, Framingham, MA, USA) at 37 °C for 18 h. The samples were desalted with C18 ZipTip (Millipore, Bedford, MA, USA) and eluted with H2O/acetonitrile (5/5; v/v). The ZipTip eluates were dried in a vacuum centrifuge. Desalted samples were rehydrated in 0.1% formic acid (FA) and were analyzed by liquid chromatography mass spectrometry (LC-MS) using a nanoLC Eksigent 400 system (Eksigent, AB Sciex), coupled online to a TripleTOF6600 mass spectrometer (AB Sciex). Peptide separation was performed using liquid chromatography with a trap and elution conﬁguration using a nano trap column (350 μm × 0.5 mm, 3 μm, 120 Å, AB Sciex) and a nano ChromXP C18 reverse phase column (75 μm × 15 cm, 3 μm, 120 Å, AB Sciex) at 300 nl/min with a 90 min linear gradient of 8-30% acetonitrile in 0.1% FA, and then, with a 10 min linear gradient of 30% to 40% acetonitrile in 0.1% FA. The mass spectrometer was operated in information-dependent acquisition (IDA) mode, scanning full spectra (400–1500 m/z) for 250 ms, followed by up to 30 MS/MS scans (100–1800 m/z for 50 ms each), for a cycle time of 1.8 s. Candidate ions with a charge state between +2 and + 5 and counts above a minimum threshold of 125 counts per second were isolated for fragmentation, and one MS/MS spectrum was collected for 12 s before adding those ions to the exclusion list. Rolling collision energy was used with a collision energy spread of 15. The mass spectrometer was operated using the Analyst TF 1.7.1 software program (AB Sciex). For data dependent acquisition (DDA, SWATH acquisition), the parameters were set as follows: 100 ms TOF MS scan, followed by 200 variable SWATH windows, each at a 50 ms accumulation time, for m/z 400–1250. MS/MS SWATH scans which were set at a 5 Da window overlapping by 1 Da for m/z 400–1250 and varied on each side of the mass range. The total cycle time was 9.6 s. A rolling collision energy (CE) parameter script was used to automatically control the CE.
