# Supplementary material for: Eukaryotic initiation factor 2 signaling behind neural invasion linked with lymphatic and vascular invasion in pancreatic cancer
Source: Sci Rep. 2021 Oct 27;11:21197. doi: 10.1038/s41598-021-00727-3 (PMC8551178; doi:10.1038/s41598-021-00727-3)
Supplement: Supplementary file 6 — Supplementary Information 6. [file 41598_2021_727_MOESM6_ESM.docx]

**Supplemental Content 4**

***Proteomics Data Analysis***

Acquired spectra were searched against the UniProt reviewed database using the Paragon algorithm embedded in the ProteinPilot software program v5.0.1 (AB Sciex, https://sciex.com), with the following search parameters: (i) sample type: identiﬁcation, (ii) Cys alkylation: iodoacetamide, (iii) digestion: trypsin, (iv) instrument: TripleTOF 6600, (v) species: Homo sapiens, (vi) ID focus: biological modiﬁcations, (vii) detected protein threshold: > 0.05 (10% conﬁdence). The detected protein threshold was set to the minimum level to enhance the number of wrong answers to enable the curve ﬁtting by an independent FDR analysis (1). This was carried out by the target-decoy approach provided with the ProteinPilot software program, which was used to assess the quality of the identiﬁcations. Positive identiﬁcations were considered to be when identiﬁed proteins and peptides reached a 1% local FDR (2). The resulting group file was loaded into Peakview v2.2.0 (AB Sciex, https://sciex.com) and peaks from SWATH runs were extracted with a peptide confidence threshold of 99% and a false discovery rate < 1%. The SWATH ﬁles were then exported to the MarkerView software program v1.3.0.1 (AB Sciex, https://sciex.com) and the peak areas of individual peptides were normalized to the sum of the peak areas of all detected peptides.

Proteomics data were visualized using Qlucore Omics Explorer v3.7 (Qlucore, New York, NY, USA, https://qlucore.com). Proteomic signatures were compared between the high-grade ne and low-grade ne groups. Differentially expressed proteins were identified using t-test with Benjamini-Hochberg correction, with a p-value cutoff set at < 0.05 and log2FC set at ≥1.

Furthermore, QIAGEN Ingenuity Pathway Analysis (QIAGEN IPA, QIAGEN Inc., Valencia, CA, USA, https://www.qiagen.com) was performed to identify canonical pathways, protein interactions, and functional networks that are most significant to our proteomics results. The core analysis was carried out with the settings of indirect and direct relationships between molecules based on experimentally observed data, and data sources were considered from mammal databases in the Ingenuity Knowledge Base. Fisher’s exact test was used to determine the probability that biological functions and/or diseases were over-represented in the protein dataset.

**References**

1. Tang WH, Shilov IV, Seymour SL. Nonlinear fitting method for determining local false discovery rates from decoy database searches. *Journal of proteome research.* 2008;7:3661-3667.

2. Sennels L, Bukowski-Wills JC, Rappsilber J. Improved results in proteomics by use of local and peptide-class specific false discovery rates. *BMC bioinformatics.* 2009;10:179.
