# Supplementary material for: Eukaryotic initiation factor 2 signaling behind neural invasion linked with lymphatic and vascular invasion in pancreatic cancer
Source: Sci Rep. 2021 Oct 27;11:21197. doi: 10.1038/s41598-021-00727-3 (PMC8551178; doi:10.1038/s41598-021-00727-3)
Supplement: Supplementary file 7 — Supplementary Information 7. [file 41598_2021_727_MOESM7_ESM.docx]

**Supplemental Content 5**

***Other Statistical Analyses***

Continuous variables were expressed as medians (ranges) and analyzed using a nonparametric method (i.e., Mann–Whitney *U*-test) because the variables were visually not normally distributed data. Categorical variables were reported as numbers (percentages) and analyzed using the chi-squared test or Fisher’s exact test, as appropriate. In multiple comparisons, results were assessed using the Kruskal-Wallis test with a Dunn-Bonferroni adjustment.

Correlations between two observed [ordinal variables](https://en.wikipedia.org/wiki/Level_of_measurement) were assessed using polychoric correlations. A correlation was considered statistically significant when the correlation coefficient (r) was beyond 0.3. Recurrence free survival (RFS) and disease specific survival (DSS) were estimated using the Kaplan–Meier method, and differences in the survival rates among the groups were compared using the log-rank test. RFS was defined as the time from the operation to the date of disease recurrence. DSS was defined as the time from the operation to the time of death due to PDAC, or the last follow-up time. This study was planned with a maximum follow-up period of five years. A difference was considered to be significant for values of P < 0.05. The statistical analyses were performed using IBM SPSS Statistics v26.0 (IBM Corp, Armonk, NY, USA, https://www.ibm.com) or R software program v4.0.5 (R Foundation for Statistical Computing, Vienna, Austria, https://www.R-project.org) (1). Data were visualized using GraphPad Prism v9.1.0 (GraphPad Software, San Diego, CA, USA, https://www.graphpad.com)

**References**

1. R Core Team. R: A Language and Environment for Statistical Computing. R Foundation for Statistical Computing 2021. https://www.R-project.org
